# Wrapping of Nano- and Microgels by Lipid-Bilayer Membranes

**DOI:** 10.1021/acsmacrolett.5c00424

**Published:** 2025-09-19

**Authors:** Tanwi Debnath, Jiarul Midya, Thorsten Auth, Gerhard Gompper

**Affiliations:** † Theoretical Physics of Living Matter, 28334 Institute for Advanced Simulation, Forschungszentrum Jülich, 52425 Jülich, Germany; ‡ Department of Physics, School of Basic Sciences, 231530 Indian Institute of Technology Bhubaneswar, Jatni, Odisha - 752050, India

## Abstract

The wrapping of nano- and microparticles is a fundamentally important pathway for their cellular uptake and depends on the physicochemical properties of both particle and membrane. Polymeric gels are a versatile class of materials whose elastic properties can be tuned in a wide range from ultrasoft to hard by changing the density of cross-linkers. Using spring networks for the microgels and triangulated surfaces for the membranes, we study microgel wrapping with computer simulations. The interplay of microgel and membrane deformation is controlled by the competition between microgel elasticity and membrane bending rigidity. Compared with hard particles, the range of adhesion strengths for which partial-wrapped states are stable is enlarged. Volume and surface area of partial-wrapped microgels can be significantly reduced compared with those of free microgels. Understanding microgel wrapping can help us to design polymeric particles for biomedical applications, e.g., as membrane markers and targeted drug delivery vectors.

## Graphical abstract

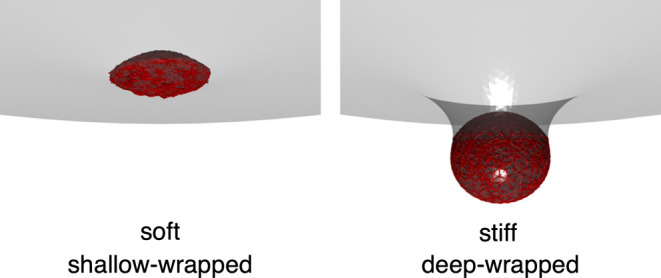

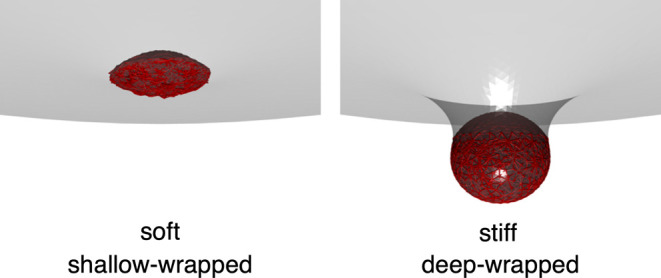

Biological cells exchange material and information with their surroundings across their plasma membranes. Endocytosis and exocytosis are well-known mechanisms to transport cargo across the lipid bilayer. For example, extracellular vesicles,^1^ filamentous viruses like Marburg and Ebola viruses,^2^ and HIV-1 virions enter cells by wrapping. Elasticity is an important physicochemical property of the particles. The mechanical properties of Murine Leukemia virus particles differ considerably in different maturation stages,^3^ and SARS-CoV-2 virions and synthetic SARS-CoV-2 miniviruses are very compliant.^4,5^ The elasticity of HIV-1 virions affects their entry into host cells.^6^ Elastic particles, such as lipid particles^7,8^ and polymeric particles like microgels,^9–11^ are designed to deliver drugs in vivo.

The physicochemical properties of polymeric gels can be tuned in a wide range,^12,13^ and their production can be scaled up to industrial levels.^14^ Stimulus sensitivity allows us to externally modify the size and elasticity of microgels: swelling and collapse can be controlled by, e.g., temperature,^15^ pH,^9^ solvent composition,^16^ and light.^17^ The interplay of the molecular architecture and the mesoscopic properties of microgels has also been studied using coarse-grained MD simulations.^18^ There are numerous applications microgels in biological and medical systems, including drug delivery vectors,^19^ scaffolds to stimulate and guide cell growth,^20^ and fostering wound healing.^21^ Microgels used for applications in cell biophysics have sizes ranging from 100 nm to 100 μm, a Poisson’s ratio of about 0.25 in the swollen state, and can have Young’s moduli in the full range of 100 Pa to 100 kPa, comparable to values reported for the elastic moduli of eukariotic cells.^22^

For elastic particles, the interplay of the deformation energy costs of the membrane that wraps the particle and the particle itself, and adhesion energy gain between the membrane and the particle, leads to more complex wrapping behavior compared to hard particles.^23–25^ Electron micrographs of soft silica nanocapsules show the deformation of the capsules upon membrane wrapping.^26^ Dissipative particle dynamics (DPD) simulations for microgel wrapping reveal the importance of cross-linker density, gel hydrophobicity/hydrophilicity, and solvent quality for the shapes of membrane-adhered nanogels and their wrapping.^27,28^ However, so far, the generic model system for in-silico studies on wrapping of elastic particles has been unilamellar vesicles.^29–31^ Microgels can serve as a model system with 3D bulk elasticity, which is fundamentally different from the curvature elasticity originating from the 2D vesicle membrane.

Here, we characterize stable states, microgel shapes, and wrapping transitions for elastic microgels at lipid-bilayer membranes. We use a spring-network model for the microgels, a triangulated-surface model for the fluid membranes, and receptor–ligand bonds to model the microgel-membrane attachment, see Figure 1. An important advantage of using continuum elasticity theory instead of molecular modeling is that the theory does not contain any intrinsic length scale. Therefore, our model is applicable to all sizes of particles, from nano- to microgels. The total energy of the system is

1 $$E_{\mathrm{tot}}=\int_{S_{\mathrm{mem}}}dS\left[2\kappa H^{2}+\sigma \right]+E_{\mathrm{mg}}-N_{\mathrm{ad}}U_{\mathrm{eff}}$$

where the first term is the curvature-elasticity Hamiltonian for the deformation energy of the lipid-bilayer membrane,^32^ the second the deformation energy of the microgel, and the third the binding energy between the microgel and the membrane. Here, *S*_mem_ is the total area, κ is the bending rigidity, and σ is the tension of the membrane. The mean curvature *H* = (*c*_1_ + *c*_2_)/2 describes the local membrane shape, where *c*_1_ and *c*_2_ are the principal curvatures at each point of the membrane. *N*_ad_ vertices of the microgel are assumed to be bound to the membrane with an effective bond energy *U*_eff_.^33^ The common microgel-membrane vertices can represent discrete microgel-substrate adhesion sites, such as receptor–ligand bonds,^34^ dangling hydrophobic polymers and specific chemical groups that adhere the microgel to a substrate,^35,36^ and screened electrostatic interactions at salt concentrations comparable to those of physiological salt solutions.^37^

**Figure 1:**
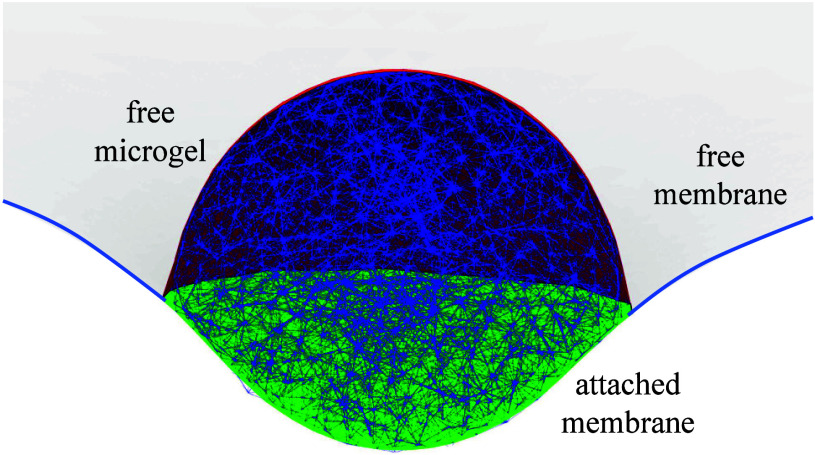
Simulation snapshot of a partial-wrapped microgel with each vertex connected with neigboring vertices by on average 18 harmonic springs. The Young’s modulus of the microgels estimated to be *Y* = 22.6 κ/*R*_mg_^3^ and the membrane is tensionless. The attached outer vertices of the microgel are also vertices of the triangulated membrane. The white and green surface model the free and attached membrane respectively; the red surface indicates the positions of the unbound outer microgel vertices.

With the help of energy minimization,^38^ we obtain the equilibrium shapes of microgels and membranes for wrapping initially spherical microgels at initially planar membranes. The microgels are constructed by 3D networks of Hookean springs with spring constant *k*_sp_, connecting *N*_V_ vertices in spheres of radius *R*_ini_. The microgel deformation energy *E*_mg_ is calculated numerically as the sum of the energies of its springs. The elastic moduli for our *in silico* microgels can be estimated to be^39^

2 $$K_{\mathrm{ana}}=\frac{k_{\mathrm{sp}}}{9V_{\mathrm{ini}}}\underset{i}{\sum }d_{0,i}^{2}$$

for the bulk modulus and

3 $$Y_{\mathrm{ana}}=3K_{\mathrm{ana}}\left(1-2\nu_{\mathrm{ana}}\right)=\frac{k_{\mathrm{sp}}}{6V_{\mathrm{ini}}}\underset{i}{\sum }d_{0,i}^{2}$$

for the Young’s modulus, where the sums run over all edges. The equilibrium spring lengths are *d*_0,*i*_ and the microgel volume is *V*_ini_. As for any spring network with only central forces between every two vertices, the Poisson’s ratio is ν = 0.25.^40^ The total energies of the microgel-membrane systems are determined for various fractions *f*_ad_ = *N*_ad_/*N*_lig_ of outer microgel vertices attached to the membrane, initially forming a compact cluster on a spherical cap, where *N*_lig_ is the total number of initially outer microgel vertices. We use the freely available software package “Surface Evolver” for the calculations;^38^ Details of the simulations and the evaluation are discussed in the SI.

The elastic properties of microgels are routinely characterized experimentally and in simulations.^12^ We simulate microgels under compression in spherical confinement, see Figure 2(a). The equilibrium free-microgel radii are smaller than those of the initial sphere, *R*_mg_ = (3*V*_mg_/(4π))^1/3^ < *R*_ini_,^18,41^ see Table 1. Our simulation shows the energy of a microgel in spherical confinement of radius *R*_sc_ < *R*_mg_ as a function of the confinement volume *V*_sc_ = 4π*R*_sc_^3^/3, see Supporting Information (SI). To calculate the bulk modulus, we then fit the energy to

4 $$E_{\mathrm{sc}}=\frac{K_{\mathrm{mg}}}{2}\frac{{\left(V_{\mathrm{mg}}-V_{\mathrm{sc}}\right)}^{2}}{V_{\mathrm{mg}}}$$

using the bulk modulus *K*_mg_ and the volume *V*_mg_ of the free microgel as fit parameters, see Table 1. The measured bulk moduli are 85–90% of those predicted by eq 2.

**Figure 2:**
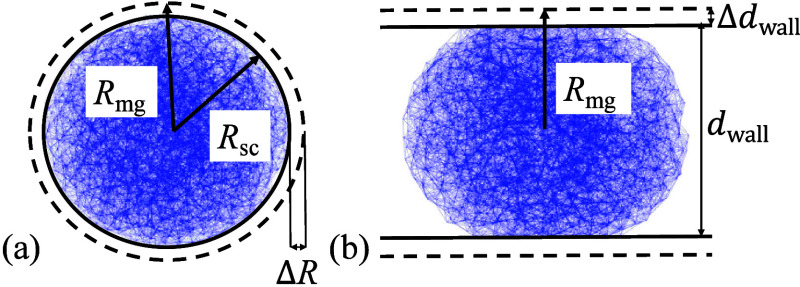
Elastic network model for a spherical microgel with *N*_v_ = 2400 vertices. (a) Sketch of a microgel confined in a sphere of radius *R*_mg_. (b) Microgel confined between two parallel planar walls at distance *d*_wall_. Simulation snapshot for Δ*d*_wall_/*R*_mg_ = (*R*_mg_ – *d*_wall_/2)/*R*_mg_ = 0.9.

**Table 1:** Bulk Moduli and Effective Radii for Microgels Modeled Using an Elastic Network Model with Various Numbers of Vertices *N*_V_ ^a^

*N*_V_	*R*_mg_/*R*_ini_	*K*_mg_ *R*_mg_/*k*_sp_	*K*_ana_ *R*_ini_/*k*_sp_
1200	0.982	11.25 ± 0.01	13.03
2400	0.985	13.07 ± 0.01	15.23
4800	0.988	18.02 ± 0.01	20.09
9600	0.990	20.79 ± 0.01	24.52

a The effective microgel radius *R*_mg_ and bulk modulus *K*_mg_ are determined from fits of the numerical data to eq 4. The analytical value *K*_ana_ has been calculated using eq 2, assuming a microgel volume *V*_ini_ = 4/3π*R*_ini_^3^. The error bars are obtained from the fits, see SI.

To determine Young’s moduli *Y* and Poisson’s ratios ν, we confine microgels with various numbers of vertices between two parallel planar walls with distance *d*_wall_ and wall shift Δ*d*_wall_ = (*R*_mg_ – *d*_wall_/2),^42^ see Figure 2b. The deformation-energy costs for confined microgels are obtained upon decreasing wall-to-wall distance from 2*R*_mg_ to *d*_wall_,^43^

5 $$E_{\mathrm{wall}}\left(d_{\mathrm{wall}}\right)=\frac{8}{15}\frac{Y_{\mathrm{Hertz}}R_{\mathrm{mg}}^{3}}{\left(1-\nu_{\mathrm{Hertz}}^{2}\right)}{\left(\frac{\Delta d_{\mathrm{wall}}}{R_{\mathrm{mg}}}\right)}^{5/2}$$

where

6 $$Y_{\mathrm{Hertz}}=3K_{\mathrm{mg}}\left(1-2\nu_{\mathrm{Hertz}}\right)$$

Using the microgel radius *R*_mg_ and the bulk modulus *K*_mg_ from Table 1, we fit eqs 5 and 6 to our numerical data with ν_Hertz_ as fit parameter, see SI, and obtain *Y*_Hertz_, see Table 2. For comparison, we provide the analytical estimate *Y*_ana_ for the Young’s modulus obtained using eq 3 and ν_ana_ = 0.25. The measured elasticities are somewhat smaller than the analytical estimate, compare ref 44.

**Table 2:** Young’s Moduli and Poisson’s Ratios Measured by Confining Microgels between Two Parallel Planar Walls for Various Numbers of Vertices *N*_V_, see Figure 2 ^a^

*N*_V_	*Y*_Hertz_ *R*_mg_/*k*_sp_	ν_Hertz_	*Y*_ana_ *R*_ini_/*k*_sp_
1200	15.05	0.28 ± 0.06	19.55
2400	18.94	0.26 ± 0.09	22.84
4800	27.01	0.25 ± 0.05	30.13
9600	36.82	0.20 ± 0.07	36.77

a The values *Y*_Hertz_ and *ν*_Hertz_ are obtained from fits of the deformation energies to eq 5 using *R*_mg_ and *K*_mg_ from Table 1 with *ν*_Hertz_ as a fit parameter. *Y*_ana_ is estimated using eq 3 considering *ν*_ana_ = 0.25. The error bars are obtained from the fits, see SI.

We find stable nonwrapped (NW) states for small bond energies, partial-wrapped (PW) states with increasing bond energies, and ‘nearly complete-wrapped’ (NCW) states for high bond energies and various dimensionless stiffness ratios *YR*_mg_^3^/(8πκ), see Figure 3; details of the calculation are discussed in the SI. In the NCW region, a wide-neck formation in the host membrane is observed for soft microgels due to the finite spring length, in contrast to the infinitesimally small neck radius for hard particles and vesicles.^30,45^ The wrapping fraction does not reach unity even at very high *U*_eff_, see SI. The wrapping transitions are calculated analogously to thermodynamic phase transitions.^24,45^ The binding transition from NW → PW at bond energy *U*_1_ is continuous without an energy barrier. For soft microgels, it occurs at significantly lower bond energies *U*_eff_ compared with the NW → CW transition at *U*_hs_ for hard spherical particles of the same size. The envelopment transition at *U*_2_ > *U*_hs_ shifts to higher effective bond energies the softer the microgel is, and is continuous for *YR*_mg_^3^/(8πκ) > 1 and discontinuous, accompanied by a shape transition,^46^ for *YR*_mg_^3^/(8πκ) ≤ 1. Whereas the microgel shapes for high *YR*_mg_^3^/(8πκ) remain almost spherical throughout the entire wrapping process, they are oblate in partial-wrapped states for small *YR*_mg_^3^/(8πκ), see Figure 3b. A facilitated engulfment of hard nanogels by lipid-bilayer membranes has also been reported using molecular simulations for nanogel-wrapping.^47^

**Figure 3:**
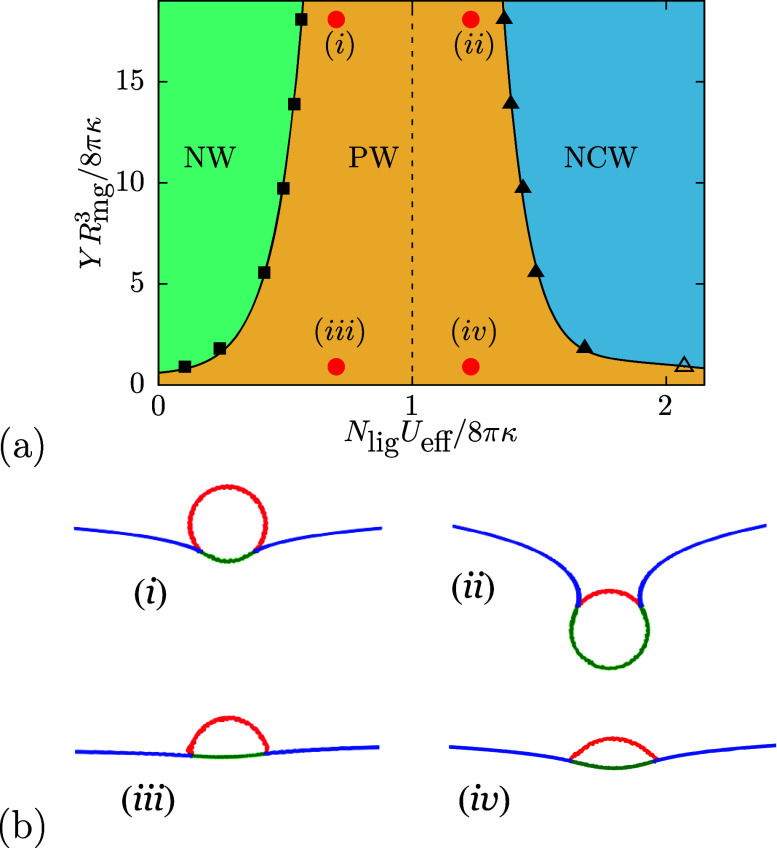
Wrapping states of microgels at fluid tensionless, initially planar membranes. The parameters for the microgel are the same as those used for Figure 2; *N*_lig_ ≈ 860 vertices have been identified as outer microgel vertices. (a) Wrapping diagram for various microgel-membrane stiffness ratios and effective bond energies *U*_eff_, showing the transition *U*_1_ between the nonwrapped and the partial-wrapped state, and the transition *U*_2_ between the partial-wrapped and the nearly complete-wrapped state. Filled symbols indicate continuous, and open symbols discontinuous transitions. The direct NW → CW transition for hard spherical particles is indicated by the dashed line. (b) Simulation snapshots for (i, ii) hard (*YR*_mg_^3^/(8πκ) = 18.08) and (iii, iv) soft (*YR*_mg_^3^/(8πκ) = 0.90) microgels at *N*_lig_ *U*_eff_/(8πκ) = 0.7 and 1.23.

Microgel-deformation energies increase with increasing adhered fractions for small *f*_ad_ and decrease for large *f*_ad_, see Figure 4a. An almost stress-free spherical shape of the microgel is recovered for high adhesion fractions. Furthermore, the maximal microgel-deformation energy remains small for both very stiff microgels that do not deform significantly, and very soft microgels, for which the deformation-energy costs are small; for 0.64 ≲ *YR*_mg_^3^/(8πκ) ≲ 1.06, the microgel deformation energy exceeds the membrane deformation energy at *f*_ad_ ≈ 0.5.

**Figure 4:**
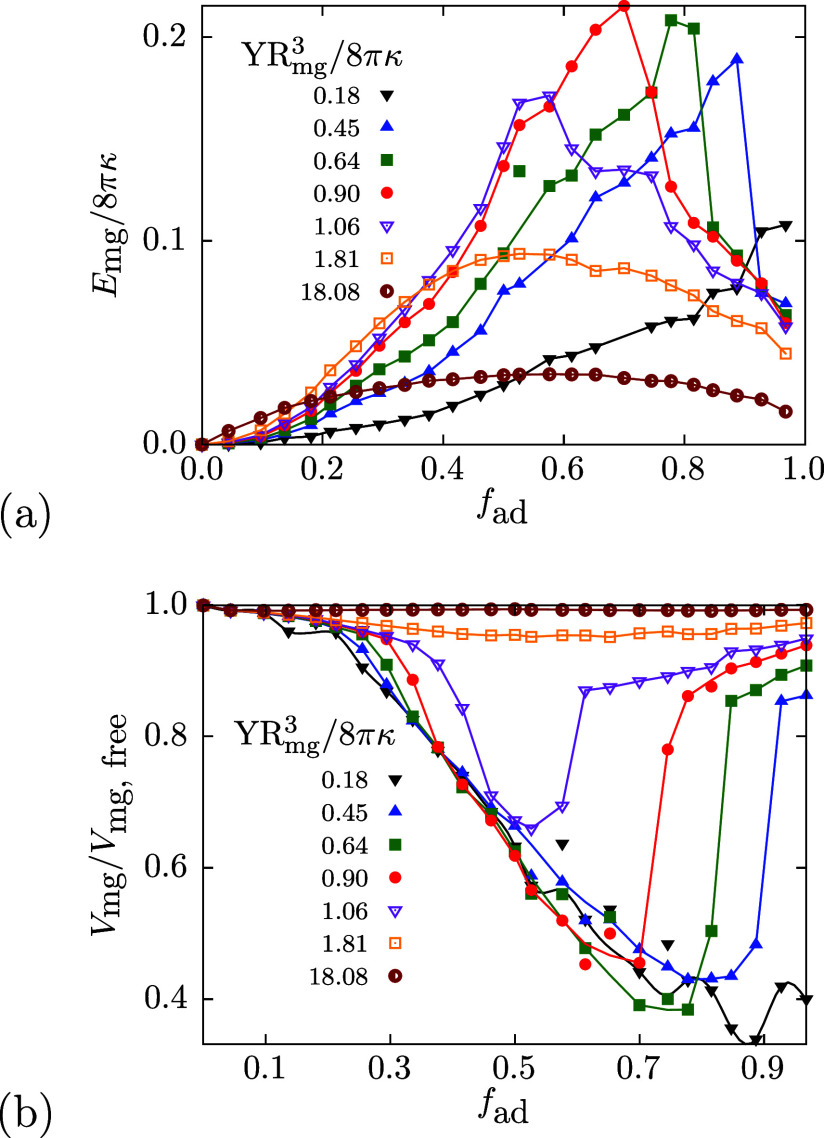
Deformation energy and volumes of microgels with various Young moduli *Y* at tensionless, initially planar membranes for various *f*_ad_. The geometrical parameters for the microgel are the same as those used for Figure 2. (a) Microgel deformation energy and (b) volume as a function of *f*_ad_.

For *YR*_mg_^3^/(8πκ) < 1, the microgel volume decreases significantly for oblate partial-wrapped states compared with those of the free, spherical microgels, see Figure 4b. The maximum decrease grows monotonically with decreasing *YR*_mg_^3^/(8πκ); for *YR*_mg_^3^/(8πκ) ≈ 0.18, the volume drops below 40% of the free microgel’s volume. The sudden increase in volume with further increasing *f*_ad_ for soft microgels coincides with the discontinuous wrapping transition from almost planar to cup-like membrane shapes at *U*_2_, see SI. For very soft microgels, we do not observe an increase in volume for large *f*_ad_, even for the highest adhesion fraction considered, *f*_ad_ = 0.96. Similarly, the surface area of microgels changes during the wrapping process, see SI.

A finite membrane tension qualitatively changes the energy landscape. For *YR*_mg_^3^/(8πκ) = 0.9 and varying σ̃ = σ*R*_mg_^2^/κ, the energy landscape features one or two step-like increases with increasing adhesion fraction, indicating that the transitions between the wrapped states are first-order transitions, see SI. The binding transition is continuous and insensitive to the membrane tension, in agreement with what is known for wrapping of hard particles.^24,48^ However, the minimal bond energy *U*_eff_ for stable nearly complete-wrapped states increases significantly with increasing tension due to the increased energy costs for enveloping a particle, see Figure 5a. A sufficiently high membrane tension leads to a triple point where three partial-wrapped states, PW I, PW II, and NCW, coexist, see Figure 5a. The triple point marks the minimal membrane tension above that a new wrapping transition between two partial-wrapped states exists with an energy barrier and a discontinuity in the adhesion fraction. The microgel shapes in the PW I state are oblate and in the PW II state cup-like; in the NCW state, the microgel shapes are almost spherical, see Figure 5b. For various microgel-to-membrane elasticity ratios, total energies, asphericities, volumes, and surface areas at membranes with finite tension are discussed in detail in the SI.

**Figure 5:**
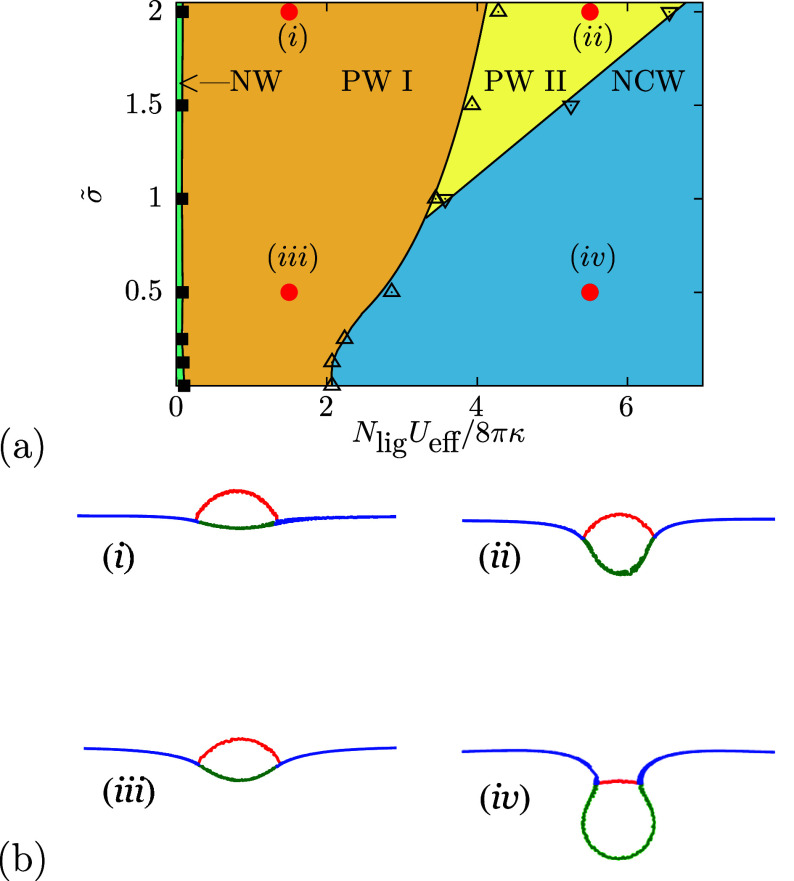
Wrapping states of microgels with various membrane tensions for microgel-to-vesicle stiffness ratio *YR*_mg_^3^/8πκ = 0.90. The parameters for the microgel are the same as those used for Figure 2. (a) Wrapping diagram for various membrane tensions and effective bond energies *U*_eff_. Filled symbols denotes continuous transitions, whereas open symbols discontinuous transitions. (b) Simulation snapshots for (i, ii) high and (iii, iv) low membrane tension at high (5.5) and low (1.5) effective bond potentials.

To conclude, we have studied the wrapping of swollen microgels at lipid-bilayer membranes. The nearly complete-wrapped states are precursor states to complete cellular uptake, for which additional biological processes are required to break the neck. Our disordered spring networks account for the disordered nature of the polymer network in an actual microgel.^49^ The number of vertices in our simulation, ranging from 2400 to 9600, is comparable to those of microgels with 1 mol % and 2 mol % of cross-linkers and swollen radii of 400–500 nm.^50^ Using the Hertz model, we extract Young’s moduli and Poisson’s ratios from simulations of microgels compressed in spherical confinement and between parallel walls. For microgels having different numbers of cross-linkers with the same Young modulus, we get similar deformation energies, which validates our model.

The wrapping behavior is controlled by the dimensionless parameter *YR*_mg_^3^/(8πκ). This parameter demonstrates that the scaling of microgel bulk compression and shear elasticity, and membrane curvature elasticity with microgel size are very different, as signaled by the volume factor. Therefore, large microgels behave very similarly to hard particles, whereas small nanogels are very deformable. For a typical lipid-bilayer bending rigidity of 50 *k*_B_ *T*, the range of Young’s moduli that we studied corresponds to 150 Pa ≤ *Y* ≤ 6 kPa for microgels with radius 250 nm, and 20 kPa ≤ *Y* ≤ 750 kPa for nanogels with radius 50 nm. Thus, our in-silico microgels span the range from ultrasoft to stiff microgels, covering the entire biologically relevant range.^51,52^

We have calculated wrapping energies for microgels at planar membranes for various microgel-membrane elasticity ratios and membrane tensions. For very soft and very hard microgels, the deformation energy of the lipid bilayer governs the deformation-energy landscapes, while for intermediate microgel deformability, the deformation energies of the microgel and the membrane are comparable. The plasma-membrane tension can be regulated by biological cells and has been proposed to orchestrate trafficking across the membrane.^53–55^ For sufficiently high membrane tension, we find an additional discontinuous transition between the deep-wrapped and the complete-wrapped state compared to tensionless membranes; the corresponding energy barrier due to the free-membrane deformation has previously been observed for spherical hard particles.^45,56^

An interesting aspect is the buckling and wrinkling of (nearly) complete-wrapped microgels, as observed in DPD simulations of nanogels.^47^ We elucidate the possibility of buckling by studying the interplay of negative membrane tension, induced by microgel-membrane adhesion, membrane bending rigidity, and microgel bulk elasticity. We estimate that buckling requires strong adhesion and soft nanogels, see SI, and expect buckling to occur at a larger adhesion than complete wrapping. For the calculation of the wrapping-state diagrams, we did not consider buckling.

Our model for spherical microgels with homogeneous elastic properties can readily be generalized to study microgels with inhomogeneous distributions of polymers and cross-linkers,^49^ hollow microgels,^57,58^ and charged microgels.^59^ In the future, we plan to study the effect of the microgel architecture on the wrapping of single microgels. Furthermore, we will characterize membrane-mediated interactions between partial-wrapped microparticles, which have been suggested to play an important role in experiments for microgels adhering to giant vesicles.^36,60^

## Supplementary Material



## Notes

Details for the spring-network model of the microgels; Energy landscapes and further information on the phase diagrams and shapes of wrapped microgels at membranes with and without tension (PDF)
