# Supplementary material for: Wrapping of Nano- and Microgels by Lipid-Bilayer Membranes
Source: ACS Macro Lett. 2025 Sep 19;14(10):1412–7. doi: 10.1021/acsmacrolett.5c00424 (PMC12548353; doi:10.1021/acsmacrolett.5c00424)
Supplement: Supplementary file 1 [file mz5c00424_si_001.pdf]

Wrapping of Nano- and Microgels by Lipid-Bilayer Membranes
Supporting Information

Tanwi Debnath,[1,*] Jiarul Midya,[1,2,†] Thorsten Auth,[1,‡] and Gerhard Gompper[1,§]

[1] *Theoretical Physics of Living Matter, Institute for Advanced Simulation, Forschungszentrum Jülich, 52425 Jülich, Germany*

[2] *Department of Physics, School of Basic Sciences, Indian Institute of Technology Bhubaneswar, Jatni, Odisha-752050, India*

S1. MODEL AND METHODS

We model swollen microgels as an elastic network of harmonic springs with finite equilibrium lengths. The springs are unstretched in the spherical microgel rest shape. This discretization approach is also referred to as a mass-spring model (MSM) [1]. The fluid lipid bilayers are discretized as dynamically triangulated surfaces, where regular membrane bond flips ensure the fluidity during major membrane shape changes [2, 3]. We minimize the energy of the microgel-membrane system using Surface Evolver [4].

We initialize a microgel-membrane system by determining the minimal-energy membrane shapes for partial-wrapped hard spherical particles with various wrapping fractions at initially planar membranes [5]. To facilitate the analysis of the final microgel shapes, we also include a triangulated surface without deformation-energy costs covering the unwrapped part of the spherical particle. The triangle sizes on the particle are carefully adjusted to be homogeneous; additional microgel vertices are then distributed within the sphere as described in subsection A. The edge lengths of the triangles on the sphere are tuned to be similar to the average distances between neighboring interior microgel vertices. All N_{lig} vertices on the spherical particle are included in the crosslinking procedure for the microgel; the membrane adhered to the spherical particle determines the N_{ad} membrane-bound outer microgel vertices.

Whereas the membrane triangulation is regular, the microgel vertices are rather homogeneously yet randomly distributed. We take into account the randomness of the vertex positions in the microgel by minimizing the energies and calculating the equilibrium microgel and membrane shapes for several initial states. The calculations for different adhesion fractions, i.e., for different hard-sphere wrapping fractions, are performed in separate simulation runs. The initial discretization of the membrane for each adhesion fraction allows us to accurately calculate membrane bending energies without requiring further bond flips after attaching the microgel. When a microgel is attached to the membrane and the minimization is performed, its springs are compressed and stretched.

To characterize the wrapping states and transitions, we analyze the total energies of the microgel-membrane systems for various fractions $f_{\mathrm{ad}} = N_{\mathrm{ad}}/N_{\mathrm{lig}}$ of outer microgel vertices attached to the membrane, where N_{ad} vertices of the microgel bind to the membrane with an effective bond energy U_{eff}. We fit the deformation energy as a function of f_{ad} to piecewise analytical functions $E_{\mathrm{def}}(f_{\mathrm{ad}})$, and minimize the total energy

$$E_{\mathrm{tot}}(f_{\mathrm{ad}}) = E_{\mathrm{def}}(f_{\mathrm{ad}}) - U_{\mathrm{eff}} f_{\mathrm{ad}} N_{\mathrm{lig}} \quad \text{(S1)}$$

with respect to f_{ad} to find the global energy minima that correspond to the stable states of the system. The fit functions are also used to locate and characterize the wrapping transitions as continuous and discontinuous [6, 7].

For the microgel-membrane simulations, we use microgels with $N_{\mathrm{v}} = 2,400$ vertices in a sphere of radius R_{ini} and microgel effective radius $R_{\mathrm{mg}} = 0.985 R_{\mathrm{ini}}$. This corresponds to an average distance $\langle d \rangle \approx 0.1218 R_{\mathrm{mg}}$ between microgel vertices, unless otherwise specified. The radius of the wire frame for the membrane patch is $R_{\mathrm{wf}} = 15 R_{\mathrm{ini}}$. All calculations are performed using Surface Evolver [4].

A. Elastic network model

To model the microgels as 3D elastic networks, vertices are distributed randomly with a minimal distance $d_{\mathrm{min}} \approx (4\pi R_{\mathrm{ini}}^3/(3 \times 2400))^{1/3}$ between each two vertices, see Fig. S1, ensuring a rather homogeneous distribution; the maximal distance for two vertices to be connected by a Hookean spring, $d_{\mathrm{max}} \approx 2 d_{\mathrm{min}}$, is chosen such that vertices near the center of the microgel have, on average, $\langle M \rangle \approx 18$ springs, see Fig. S2. For $\langle M \rangle \gtrsim 18$, the deviation of the measured elastic modulus from the theoretical estimates in Eqs. (2) and (3) of the main text has been found in bulk not to depend on the exact number of connections [1]. Consequently, we expect vertices near the surface of the microgel to have, on average, $\langle M \rangle \approx 9$ springs.

The probability distribution for the number of springs per vertex shows that for our standard microgel with $N_{\mathrm{v}} = 2,400$ vertices, each vertex is connected by at least five springs with neighbouring vertices, see Fig. S2. By

* t.debnath@fz-juelich.de
† jmidya@iitbbs.ac.in
‡ t.auth@fz-juelich.de
§ g.gompper@fz-juelich.de

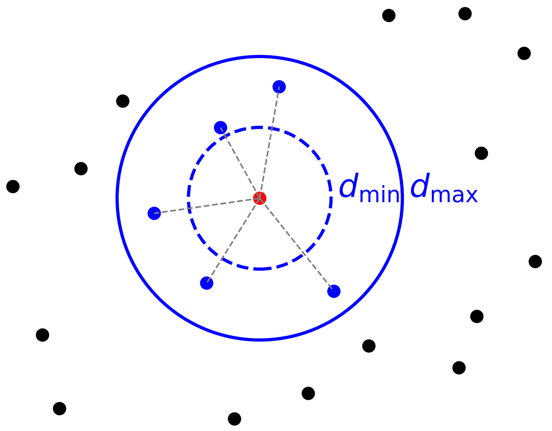

FIG. S1. Elastic network model for a spherical microgel. Sketch of a microgel with N_v vertices randomly distributed within a sphere of radius R_{ini}. The vertices have a minimum distance d_{min} to their nearest neighbour, and are connected via springs with all neighbouring vertices within a spherical shell of radius d_{max}, which act as Hookean springs.

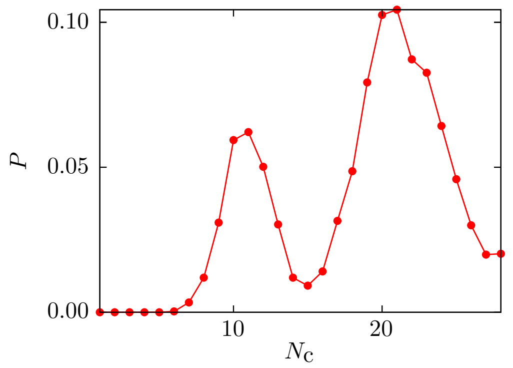

FIG. S2. Probability distribution of connections per vertex for microgels partially attached at a membrane with attachment fraction $f_{ad} = 0.5$.

construction, the maximal number of springs per vertex is limited to 28. The most probable number of springs per vertex is 21, which holds for interior vertices, and 11 for vertices near the surface. The vertices with the least number of connections are the membrane vertices that are included in the microgel crosslinking procedure and also serve as receptor-ligand bonds.

For undeformed microgels, the probability distribution for the spring lengths shows a quadratic increase with increasing spring length, $P_L \propto L_{edge}^2$, as expected for homogeneously distributed vertices, see Fig. S3. The two-shell approach for constructing the microgels, with a minimal distance between each two vertices and the maximal spring lengths, is reflected in the spring-length distribution. Systematic deviations from the quadratic increase of the histogram columns, in particular the existence of springs with lengths shorter than d_{min}, originate from

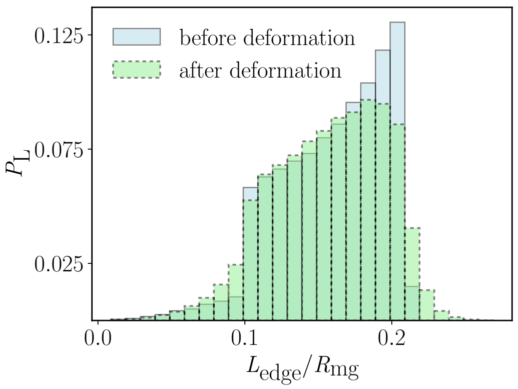

FIG. S3. Probability distribution for the spring lengths of microgels at initially planar membranes for undeformed/frozen (blue) and deformed (green) microgels and $f_{ad} = 0.5$ and microgel-to-membrane stiffness ratio $YR_{mg}^3/(8\pi\kappa) = 0.90$.

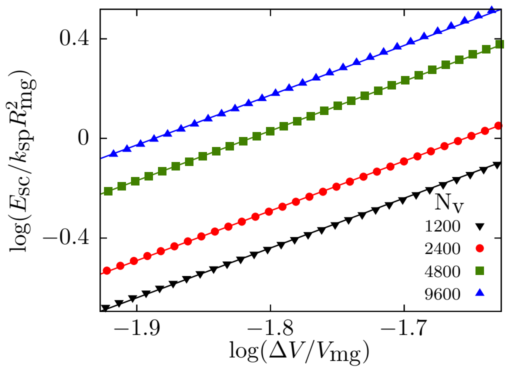

FIG. S4. Microgel deformation energies for various numbers of vertices N_V as a function of $\Delta V/V_{mg} = (V_{mg} - V_{sc})/V_{mg}$. Numerical data (symbols) along with a fit using Eq. (4) of the main text (line).

(i) the finite size of the microgel and (ii) the membrane vertices included in the microgel crosslinking procedure. In the minimal-energy state, for half-wrapped microgels the spring-length distribution widens, as some springs are stretched and others compressed.

B. Elastic properties of microgel

Figure S4 features the energy of a microgel with various numbers of vertices in spherical confinement of radius R_{sc} as a function of the confinement volume V_{sc}. To avoid the system from getting stuck in metastable states, it is key to iteratively decrease the confinement size in small steps and minimize the energy after every step, compare Ref. [8]. Starting with confinement radius $R_{sc} = R_{ini}$, we decrease R_{sc} in steps of $\Delta R = 0.001 R_{ini}$. To measure bulk modulus K, we study microgels with various numbers of vertices compressed in a sphere of ra-

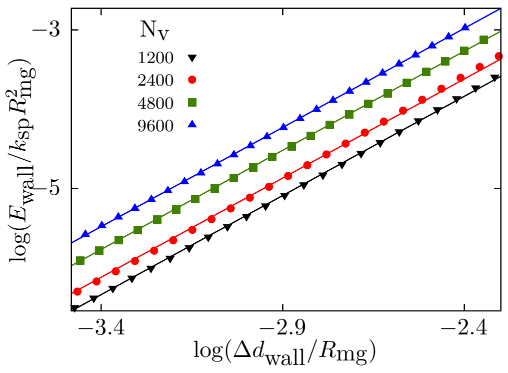

FIG. S5. Microgel deformation energies for various numbers of vertices N_V as functions of $\Delta d_{\rm wall}/R_{\rm mg}$. Numerical data (symbols) along with fits using Eq. (5) of the main text (lines).

dius $R_{\rm ini}$, corresponding to different spring lengths, and $k_{\rm sp}/(6V_{\rm ini})\sum_i d_{0,i}^2 = 21.7$. The larger the number of vertices, the closer $R_{\rm mg}$ is to $R_{\rm ini}$.

We then confine the microgels between two parallel planar walls with distance $d_{\rm wall}$ to calculate Young's moduli Y and Poisson's ratios ν. The Hertz model predicts the resulting repulsive force between the walls to be [9]

$$F_{\rm wall}(d_{\rm wall}) = \frac{4}{3}\frac{Y_{\rm Hertz}}{(1-\nu_{\rm Hertz}^2)} R_{\rm mg}^{1/2}\left(R_{\rm mg} - \frac{d_{\rm wall}}{2}\right)^{3/2} \tag{S2}$$

Integrating the force upon decreasing wall-to-wall distance from $2R_{\rm mg}$ to $d_{\rm wall}$, the deformation-energy is obtained for confined microgels with various number of vertices, see Eq. (5) in the main text and Fig. S5. The error bars in Tables 1 and 2 in the main text indicate the asymptotic standard errors from fitting the data shown in Figs. S4 and S5.

For a regular cubic spring network with lattice constant a and in total 18 nearest- and next-nearest-neighbor connections, the elastic properties are described by the Young's modulus $Y_{\rm cubic} = 5k_{\rm sp}/(2a)$, and the bulk modulus $K_{\rm cubic} = 5k_{\rm sp}/(3a)$ [1, 10, 11].

S2. WRAPPING MICROGELS AT TENSIONLESS MEMBRANES

A. Wrapping-energy landscapes

The total deformation energy of the microgel-membrane systems monotonically increases with increasing fraction $f_{\rm ad}$ of membrane-bound outer microgel vertices, see Fig. S6(a). The more deformable the microgel, the lower the total deformation energy for a given $f_{\rm ad}$ [5, 12]. Whereas the deformation energies smoothly increase with increasing wrapping fraction for stiff microgels, the increase is jump-like with increasing $f_{\rm ad}$ for soft microgels, accompanied by a microgel shape change from almost planar to cup-like, see Fig. S6(c). For $YR_{\rm mg}^3/(8\pi\kappa) \approx 0.64$ the jump occurs at $f_{\rm ad} \approx 0.6$ and shifts to higher adhesion fractions with increasing microgel deformability, see Fig. S6(a). Furthermore, our data shows that $E_{\rm def}/(8\pi\kappa) < 1$ for $f_{\rm ad} \to 1$, indicating that the wrapping remains incomplete even for high values of $U_{\rm eff}$.

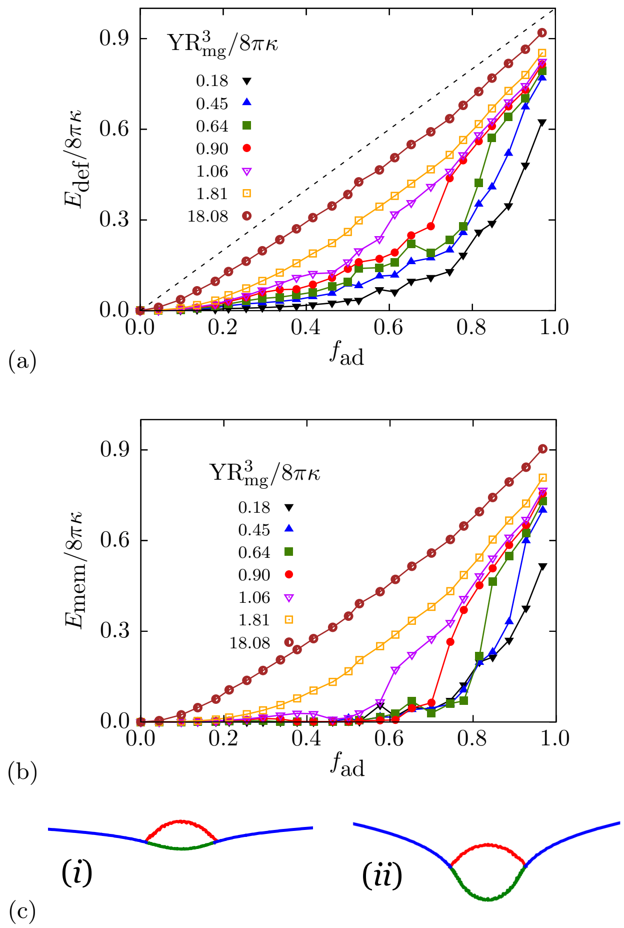

FIG. S6. Wrapping energy landscapes for microgels with various Young moduli at tensionless, initially planar membranes. The geometrical parameters for the microgel are the same as those used for Fig. 2; $N_{\rm lig} \approx 860$ vertices have been identified as outer microgel vertices. (a) Total deformation energy, (b) membrane deformation energy, and (c) shapes of oblate (*i*) and cup-like (*ii*) microgels for $YR_{\rm mg}^3/(8\pi\kappa) = 0.90$.

Because of the interplay of microgel and membrane deformations, the total-energy landscapes significantly depend on the elasticity ratios $YR_{\rm mg}^3/(8\pi\kappa)$ of microgel and membrane. Like the total deformation energies, the membrane-deformation energies monotonically increase with increasing adhesion fraction, see Fig. S6(b). Jumps to higher energies with increasing $f_{\rm ad}$ are observed for soft microgels. We calculate total energy $E_{\rm tot}$ for micro-

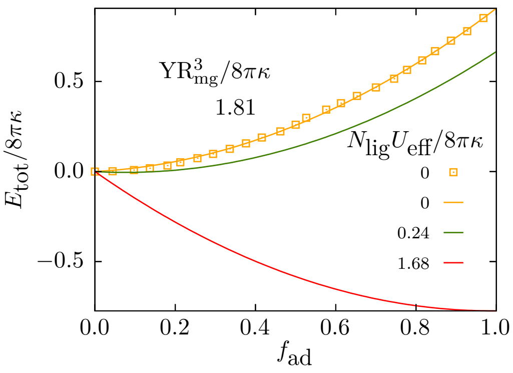

FIG. S7. Total energies of a microgel-membrane system as a function of the fraction f_{ad} of bound ligands for a tensionless membrane. The energies at the transitions are obtained using Eq. (S1) for various values of the effective receptor-ligand bond potential U_{eff}. The symbols represent the numerically calculated total deformation energies for $U_{\mathrm{eff}} = 0$, and the corresponding line is piecewise fits with second-order polynomials. The lines for finite bond potentials correspond to the binding and envelopment transitions.

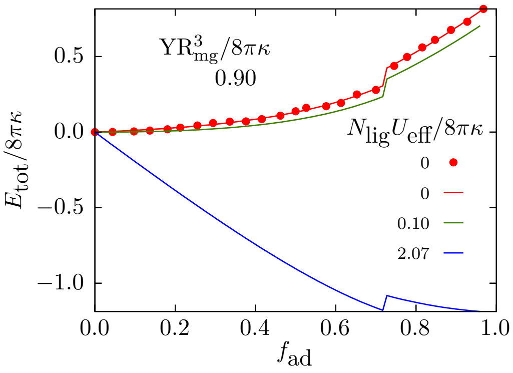

FIG. S8. Total energies of a microgel-membrane system as a function of the fraction f_{ad} of bound ligands for a tensionless membrane. The energies at the transitions are obtained using Eq. (S1) for various values of the effective receptor-ligand bond potential U_{eff}. The symbols represent the numerically calculated total deformation energies for $U_{\mathrm{eff}} = 0$, and the corresponding line is fits with Eq. (S4). The lines for finite bond potentials correspond to the wrapping transitions.

gels attached to tensionless membranes as a function of the fraction f_{ad} of adhered outer microgel vertices. The total energy is analyzed analogously to previous studies of wrapping non-spherical particles [5, 6].

For stiff microgels with microgel-to-membrane stiffness ratio $YR^3_{\mathrm{mg}}/(8\pi\kappa) = 1.81$, the energy landscape is smooth, see Fig. S7. We fit the deformation energy as a function of f_{ad} using a second-order polynomial,

$$E_{\mathrm{def}}(f_{\mathrm{ad}}) = af_{\mathrm{ad}} + bf^2_{\mathrm{ad}} \tag{S3}$$

and add the adhesion energy analytically. The binding

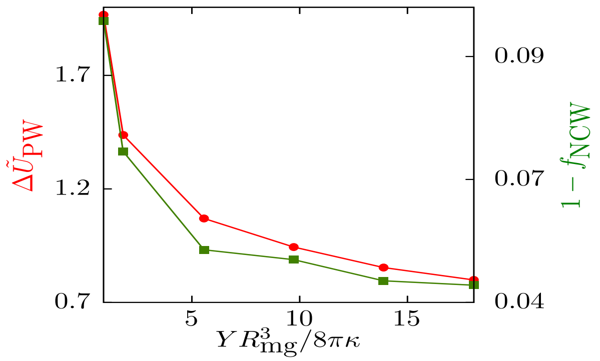

FIG. S9. Width of partial wrapped states (red) and closeness to completely wrapped states (green) of microgels for various microgel-to-membrane stiffness ratios at tensionless membranes.

and envelopment transitions are obtained using Eq. (S1) by determining the values of the effective receptor-ligand bond energies U_{eff} for that the slope $\partial E_{\mathrm{tot}}/\partial f_{\mathrm{ad}}$ vanishes at adhesion fractions $f_{\mathrm{ad}} = 0$ and 0.96, respectively. The binding transition between the non-wrapped (NW) and a partial-wrapped (PW) state takes place at $U_1 N_{\mathrm{lig}}/(8\pi\kappa) = 0.24$, and the envelopment transition between the PW and the nearly complete wrapped (NCW) state at $U_2 N_{\mathrm{lig}}/(8\pi\kappa) = 1.68$.

For soft microgels with $YR^3_{\mathrm{mg}}/(8\pi\kappa) = 0.90$, we found a combination of continuous and discontinuous transitions, see Fig. S8. The deformation energy is fitted as a function of f_{ad} with the piecewise analytical function

$$E_{\mathrm{def}}(f_{\mathrm{ad}}) = \begin{cases} a\exp(bf_{\mathrm{ad}}) - a & \text{for } f_{\mathrm{ad}} < 0.73 \\ af_{\mathrm{ad}} + bf^2_{\mathrm{ad}} & \text{for } f_{\mathrm{ad}} \geq 0.73\,. \end{cases} \tag{S4}$$

The continuous binding transition between the NW and PW state is obtained at $N_{\mathrm{lig}}U_1/(8\pi\kappa) = 0.10$ where the slope of the total energy at adhesion fraction $f_{\mathrm{ad}} = 0$ vanishes. The discontinuous envelopment transition between the PW and NCW state at $N_{\mathrm{lig}}U_2/(8\pi\kappa) = 2.07$ is found for the effective bond energy for that the value of the energy at the total-energy minimum at $f_{\mathrm{ad}} \approx 0.7$ equals the value of the energy at the highest adhesion fraction, $f_{\mathrm{ad}} = 0.96$.

B. Range of bond energies for stable partial-wrapped states

Figure S9 shows the range of effective bond energies for which the partial-wrapped states are stable and the unwrapped fraction of the microgels in the nearly complete-wrapped states as functions of the microgel-to-membrane stiffness ratio. Thus, with increasing $YR^3_{\mathrm{mg}}/(8\pi\kappa)$, the range in effective bond potential for the partially

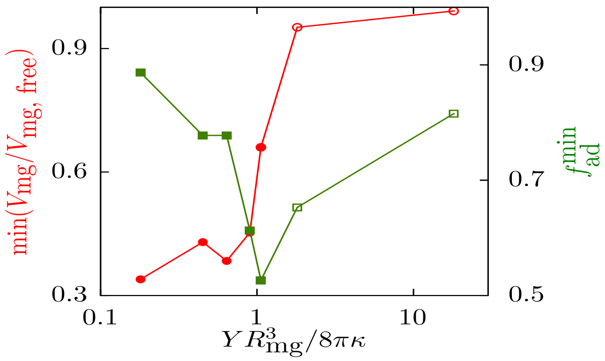

(a)

FIG. S10. Minimal volumes V_{mg} of the microgel (red) and corresponding wrapping fractions f_{ad} (green) for various microgel-to-membrane stiffness ratios. Open symbols indicate microgels with discontinuous, and closed symbols microgels with continuous envelopment transitions.

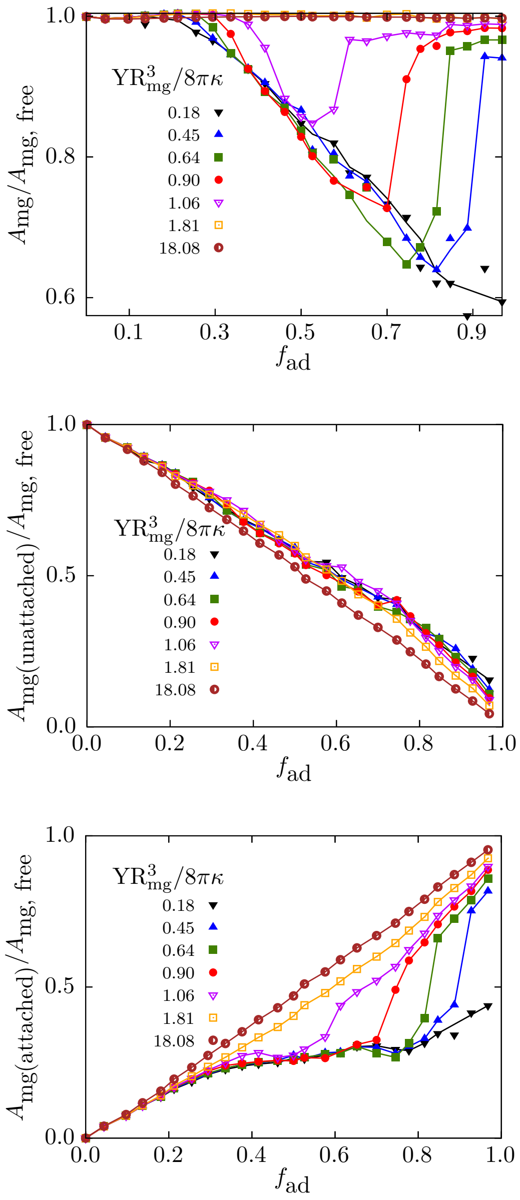

(a)

(b)

(c)

FIG. S11. Shape deformation of a microgel with various Young moduli for wrapping at a tensionless membrane: microgel (a) unattached and (b) attached surface area as a function of the fraction of bound outer vertices.

wrapped states monotonically decreases, see Fig. 3(a) of the main text, and the wrapping fraction of the nearly completely wrapped state increases.

C. Minimal microgel volumes

The transitions between stable oblate and cup-like microgel shapes are prominently visible in both the total energy and the microgel volume, see Figs. 4(b) of the main text and S6(a). For soft microgels with discontinuous envelopment transitions, we find the globally minimal volume for oblate-shaped, partial-wrapped microgels. The minimal volume decreases and the adhesion fraction for the minimum increases with increasing microgel-to-membrane stiffness ratio, see Fig. S10. For hard microgels with continuous envelopment transitions, the minimal volume is considerably smaller than for soft microgels, and the minimal volume and the adhesion fraction of the minimum both increase with increasing microgel stiffness.

D. Microgel surface areas

During wrapping, the total surface area of the microgel changes non-monotonically as the adhesion fraction increases, see Fig. S11(a). For $YR_{mg}^3/(8\pi\kappa) < 1$, the microgel surface area decrease significantly for oblate partial-wrapped states compared with those of the free, spherical microgels. The maximum decrease grows monotonically with decreasing $YR_{mg}^3/(8\pi\kappa)$; for $YR_{mg}^3/(8\pi\kappa) \approx 0.18$, the area drops below 60% of the free microgel's area. The sudden increase in area with further increasing f_{ad} for soft microgels coincides with the discontinuous wrapping transition from almost planar to cup-like membrane shapes, see Fig. S6(c). For very soft microgels, we do not observe an increase in area, even for the highest adhesion fraction we studied, $f_{ad} = 0.96$.

For partial-wrapped states, the free surface area of the microgels increases with increasing microgel deformability compared with stiff microgels, see Fig. S11(b). The attached area of microgels increases linearly for small f_{ad}; it remains almost constant for $f_{ad} \approx 0.5$, and increases steeply to the final value in the NCW state at the envelopment transition, see Fig. S11(c). The microgels with $YR_{mg}^3/(8\pi\kappa) = 0.18$ show no discontinuous transition and an NCW state with a much smaller attached area than the stiffer microgels.

E. Microgel asphericities

Throughout the wrapping process, we follow the positions of the initially outer microgel vertices. These vertices are employed to calculate the asphericity [13–15]

$$\alpha = \frac{1}{2}\frac{(\lambda_1-\lambda_2)^2+(\lambda_2-\lambda_3)^2+(\lambda_3-\lambda_1)^2}{(\lambda_1+\lambda_2+\lambda_3)^2}\,, \quad \text{(S5)}$$

where the λ_i denote the eigenvalues of the gyration tensor, which describes the microgel shape and is nonzero for all partially adhered states, $0 < f_{\rm ad} < 1$, see Fig. S12(a). For stiff microgels with $YR_{\rm mg}^3/(8\pi\kappa) \gtrsim 1.81$, the asphericities are small, $\alpha \lesssim 0.02$, for all adhesion fractions $f_{\rm ad}$; the microgels remain almost spherical throughout the wrapping process. For soft microgels, the asphericities show a peak at $f_{\rm ad} \approx 0.7$, which reflects their oblate shapes with the microgels spread on the membrane. The cylindrically symmetrical, oblate microgel shapes are reflected in two eigenvalues being approximately equal and larger than the third eigenvalue, $\lambda_1 < \lambda_2 = \lambda_3$, see Fig. S12(b-d). Because non-ellipsoidal microgel shapes are not directly considered in the ellipsoidal approximation for calculating the asphericity, clear signatures in the asphericity like the two peaks for $YR_{\rm mg}^3/(8\pi\kappa) \gtrsim 1.06$, do not necessarily indicate wrapping and microgel-shape transitions.

S3. WRAPPING MICROGELS AT MEMBRANES WITH TENSION

A. Wrapping-energy landscapes

The total microgel-membrane deformation energy remains unchanged or increases with increasing membrane tension, see Fig. S13. For small adhesion fractions, we measure the same total deformation energies for $\tilde{\sigma} = 1$ and $\tilde{\sigma} = 2$. The range of $f_{\rm ad}$ where the total deformation energies do not depend on the tension extends to higher $f_{\rm ad}$ for smaller microgel-to-membrane deformability ratios. In this regime, the membrane remains almost flat, and the microgel's contribution to the total deformation energy dominates. For $YR_{\rm mg}^3/(8\pi\kappa) = 0.9$, the energies deviate only for adhesion fractions above the discontinuous wrapping transition from oblate to cup-like, which occurs at $f_{\rm ad} \approx 0.7$, similarly as for tensionless membranes, compare Fig. S6(a). We observe a second discontinuous wrapping transition for $\tilde{\sigma} = 2$ from a cup-like to a nearly spherical shape at $f_{\rm ad} \approx 0.8$, which we attribute to an energy barrier originating from the free-membrane deformation energy as for the wrapping of hard spherical particles [5, 12].

For stiff microgels, the membrane bending energy depends almost linearly on the adhesion fraction, see Fig. S13(b), as for wrapping hard spherical particles at tensionless membranes [5, 12]. A very small membrane bending energy indicates that the membrane remains al-

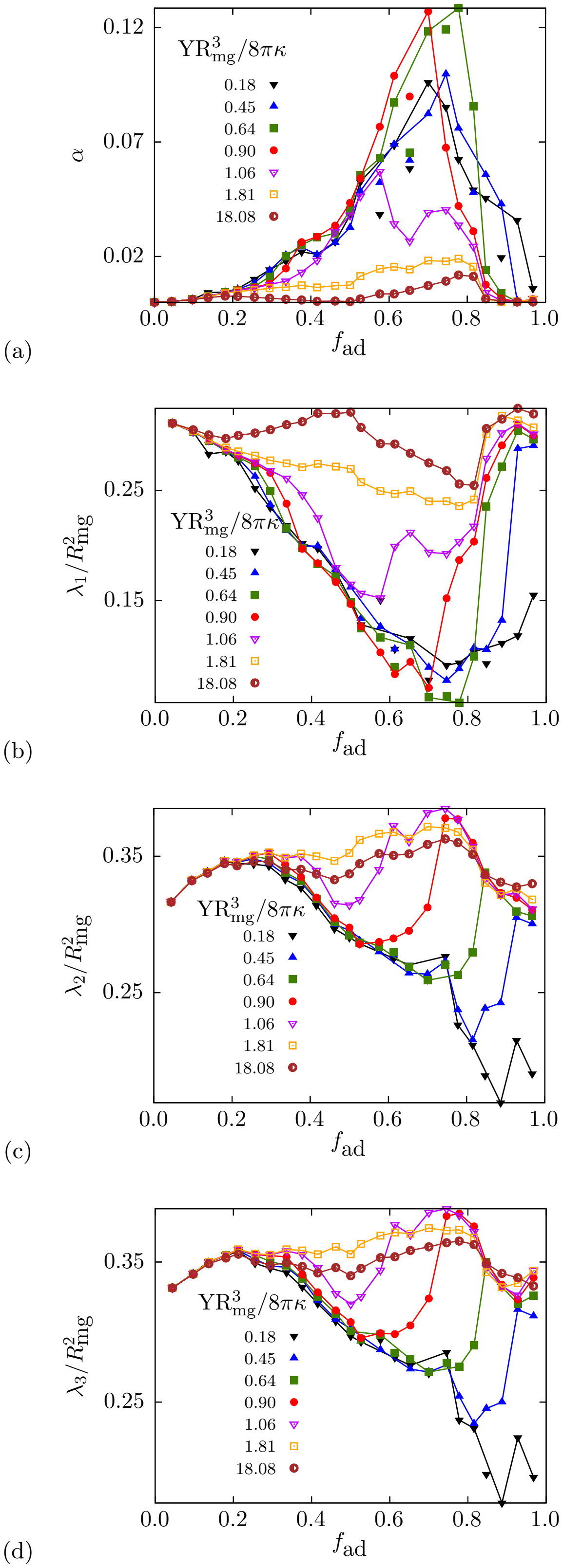

FIG. S12. Shapes of vesicles that are partially wrapped at tensionless membranes: (a) asphericity, and eigenvalues (b) λ_1, (c) λ_2, (d) λ_3, calculated using Eq. (S5).

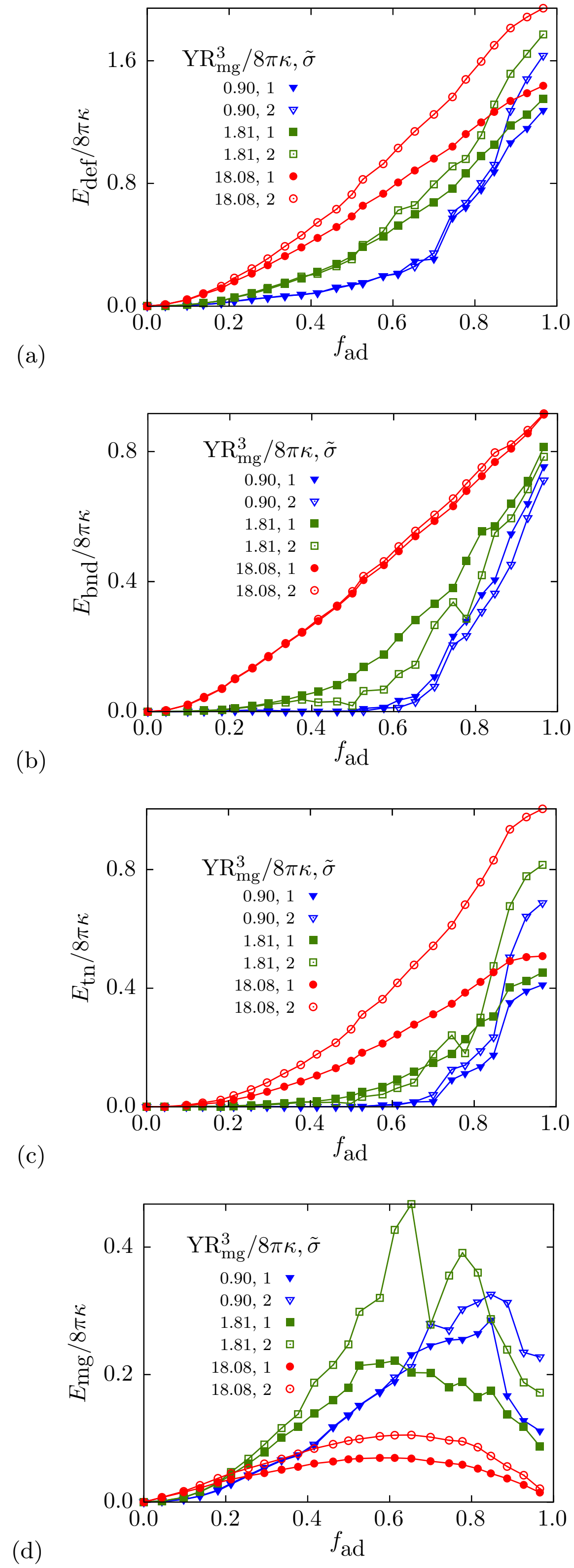

FIG. S13. Wrapping energy landscapes for microgels with various Young moduli at initially planar membranes with tension: (a) total-deformation, (b) membrane-bending, (c) membrane-tension, and (d) microgel-deformation energy as a function of the fraction f_{ad} of bound outer vertices.

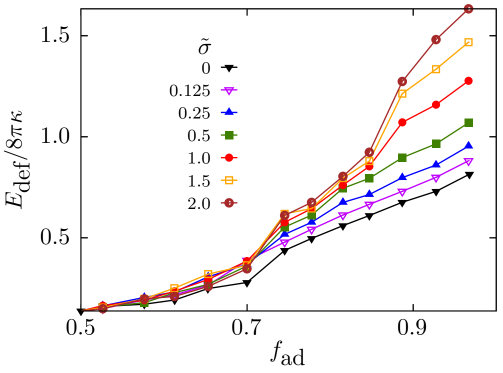

FIG. S14. Wrapping states of microgels with various membrane tensions for microgel-to-vesicle stiffness ratio $YR^3_{mg}/8\pi\kappa = 0.90$. The parameters for the microgel are the same as those used for Fig. 2. (a) Wrapping energy landscapes for various membrane tensions as a function of the fraction of bound outer vertices.

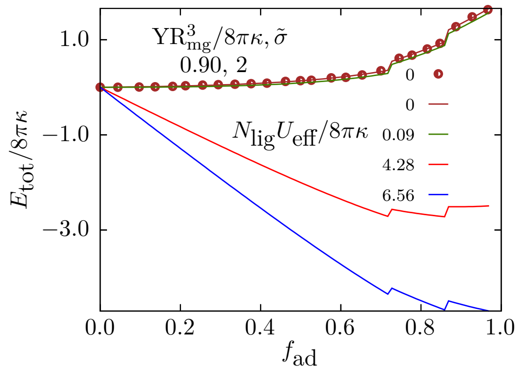

FIG. S15. Total energies of a microgel-membrane system as a function of the fraction f_{ad} of bound ligands for $YR^3_{mg}/(8\pi\kappa) = 0.90$ and membrane tension $\tilde{\sigma} = 2$. The energies at the transitions are obtained using Eq. (S1) for various values of the effective receptor-ligand bond potential U_{eff}. The symbols represent the numerically calculated total deformation energies for $U_{eff} = 0$, and the corresponding line is the fit with a piecewise function in Eq. (S6).

most planar. For soft microgels, the membrane deformation energies increase strongly with increasing adhesion fraction above the discontinuous wrapping transition. The dependence of the membrane tension energies on f_{ad} is qualitatively similar, but shows the characteristic convex shape at $f_{ad} \approx 0.9$, see Fig. S13(c), which is known from wrapping hard spherical particles [5, 12].

The microgel elastic energies vanish for $f_{ad} \to 0$, peak for intermediate f_{ad}, and decrease with increasing f_{ad} for almost completely adhered states, see Fig. S13(d). For $YR^3_{mg}/(8\pi\kappa) = 18.08$, the maximum of the microgel deformation energy is shifted to higher f_{ad} compared with the maximum for tensionless membranes. In contrast, for the two microgels with higher deformabilities, the

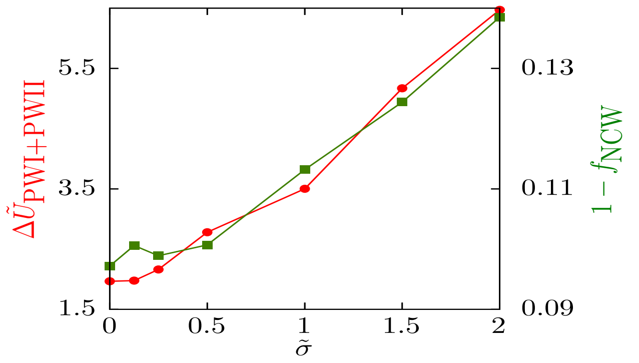

FIG. S16. Width of partial wrapped states (red) and closeness to completely wrapped states (green) of microgels for $YR_{\text{mg}}^3/(8\pi\kappa) = 0.90$ at membranes with tension.

maxima appear at similar f_{ad}, compare Fig. 4(a) of the main text. In general, the microgel deformation energy is higher compared to the tensionless membrane systems. Furthermore, for $\tilde{\sigma} = 2$, the maximum microgel deformation energy is higher than those for stiffer microgels that do not deform much and for more deformable microgels that do not require much energy to deform.

B. Wrapping transitions for various membrane tensions

The total deformation energies for $YR_{\text{mg}}^3/(8\pi\kappa) = 0.9$ and varying $\tilde{\sigma}$ increase monotonically with increasing adhesion fraction, see Fig. S14. At low tension, they show one discontinuous step-like energy increase due to the oblate to cup-like shape transition. We observe two step-like energy increases at high tension, from oblated to cup-like and from cup-like to almost spherical, because of an additional energy barrier caused by deforming the free membrane at finite tension.

Figure S15 shows the energy landscapes for the effective bond potentials at the wrapping transitions for a system $YR_{\text{mg}}^3/(8\pi\kappa) = 0.90$ with $\tilde{\sigma} = 2$. The transitions are calculated analogously to those for the tensionless membranes, compare subsection S2.A. The total deformation energy is fitted as a function of f_{ad} using the piecewise analytical function

$$E_{\text{def}}(f_{\text{ad}}) = \begin{cases} a\exp(bf_{\text{ad}}) - a & \text{for } f_{\text{ad}} < 0.73 \\ af_{\text{ad}} + bf_{\text{ad}}^2 & \text{for } 0.73 \le f_{\text{ad}} < 0.84 \\ af_{\text{ad}} + bf_{\text{ad}}^2 & \text{for } f_{\text{ad}} \ge 0.84\,. \end{cases} \tag{S6}$$

We find a continuous binding transition between the NW and PW I state at $N_{\text{lig}}U_1/(8\pi\kappa) = 0.09$. A discontinuous wrapping transition between the two partial-wrapped states PW I and PW II is found at $N_{\text{lig}}U_2/(8\pi\kappa) = 4.28$, where the total energies of the local minima for

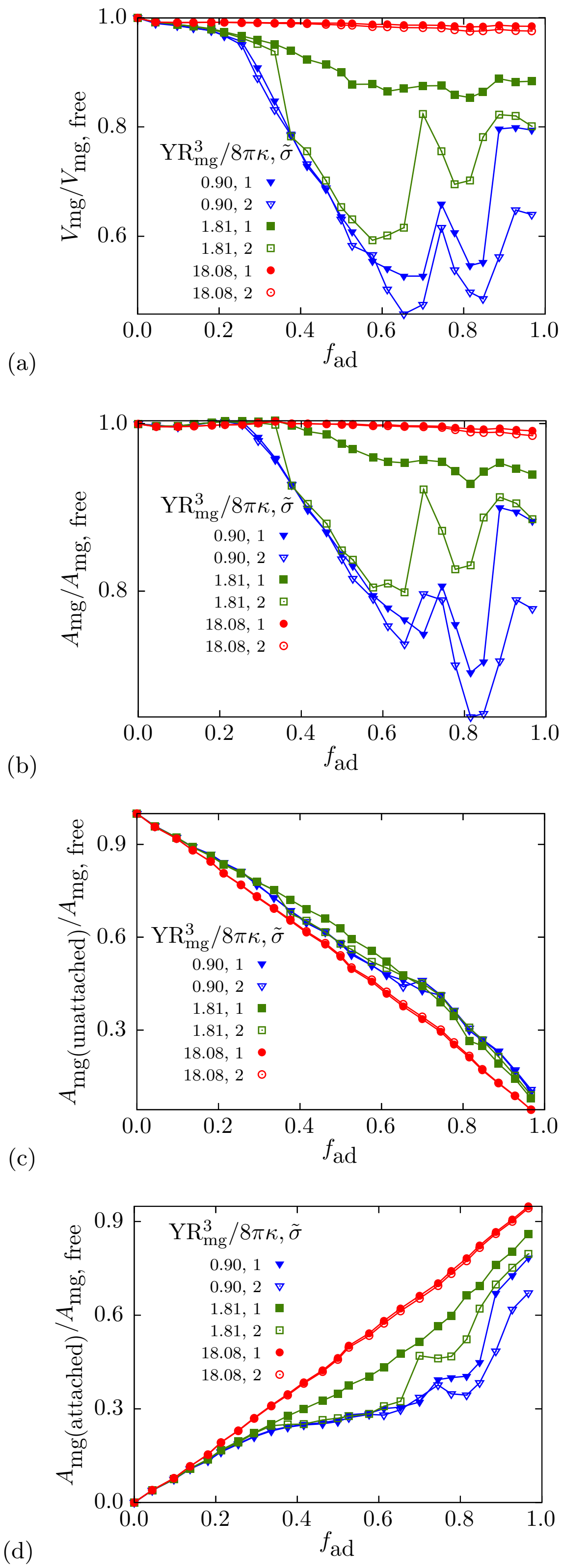

FIG. S17. Volumes and surface areas of microgels with various Young moduli at initially planar membranes with tension: (a) microgel volume, and (b) total, (c) unattached, and (d) attached surface area as functions of the fraction f_{ad} of bound outer vertices.

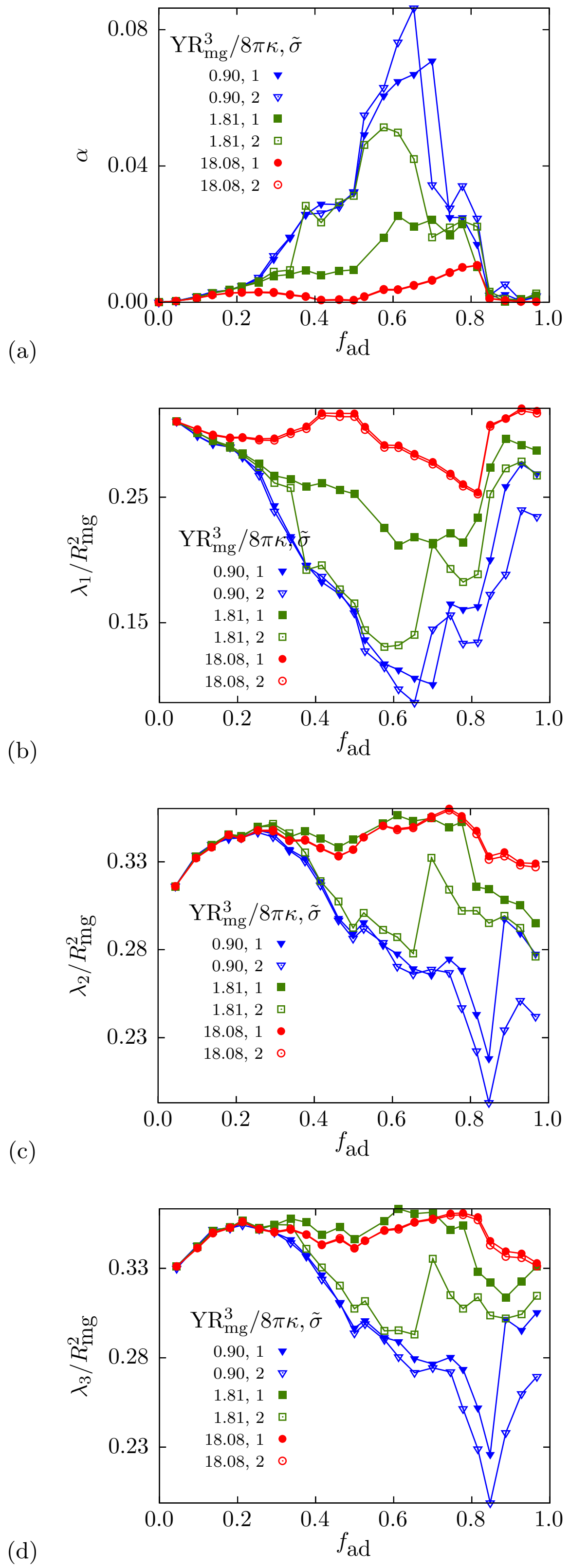

FIG. S18. Shapes of vesicles that are partially wrapped at membranes with tension: (a) asphericity, and eigenvalues (b) λ_1, (c) λ_2, (d) λ_3, calculated using Eq. (S5).

the two partial-wrapped states are equal. A discontinuous envelopment transition between the PW II and NCW states corresponds to effective adhesion strength $N_{\mathrm{lig}}U_3/(8\pi\kappa) = 6.56$. Here, the local minimum has the same energy as at $f_{\mathrm{ad}} = 0.96$.

C. Range of bond energies for stable partial-wrapped states

Figure S16 shows the range of effective bond energies for which the partial-wrapped states are stable, which are significantly larger compared to systems with tensionless membranes. The unwrapped fraction of the microgel surface area in the NCW state also increases with increasing tension, but remains small approximately 10%, for $0 \lesssim \tilde{\sigma} \lesssim 2$. For $\tilde{\sigma} \gtrsim 0.5$, both the range for effective bond energies for stable partial-wrapped states and the fraction of the unwrapped microgel surface area in the nearly complete-wrapped states increase linearly with increasing tension.

D. Microgel volumes and surface areas

The microgel volume and surface area are reduced for partially wrapped states compared with the free state, see Fig. S17(a,b). For higher microgel-to-membrane deformability ratios, both the volume and the total surface area show clear signatures of the shape transitions and increase near the transitions between oblate, cup-like and almost-spherical partial-wrapped states. Analogously to tensionless-membrane systems, compare Fig. S11, the area of the free microgel surface increases with microgel deformability and the area of the membrane-attached microgel surface decreases with increasing microgel deformability, see Fig. S17(c,d). Whereas the area of the free microgel surface area is independent on the membrane tension, for $YR^3_{\mathrm{mg}}/(8\pi\kappa) = 0.90$ and 1.81, an increased membrane tension further decreases the area of the membrane-adhered microgel surface.

E. Microgel asphericities

Figure S18(a) shows the microgel asphericities, calculated using Eq. (S5). The small asphericities for very stiff microgels with $YR^3_{\mathrm{mg}}/(8\pi\kappa) = 18.08$ indicate almost spherical shapes for all wrapping fractions. For soft microgels, the two maxima correspond to the states in-between the two discontinuous transitions between oblated and cup-like state at $f_{\mathrm{ad}} \approx 0.6$ and the cup-like and the almost spherical state at $f_{\mathrm{ad}} \approx 0.8$. As for the asphericities of microgels attached to tensionless membranes, $\lambda_1 < \lambda_2 = \lambda_3$ indicate that the ellipsoids of inertia used to characterize the shapes are oblate with

rotational symmetry, see Fig. S18(b-d).

S4. BUCKLING INSTABILITY OF A WRAPPED MICROGEL – INTERPLAY OF NEGATIVE MEMBRANE TENSION, BENDING RIGIDITY, AND MICROGEL ELASTICITY

Buckling of the membrane around complete-wrapped nanogels has been observed in dissipative particle dynamics (DPD) simulations [16]. For strong membrane adhesion, the membrane buckles to maximise the contact area between the membrane and the nanogel. The lengthscale at which the spherical surface conformation becomes unstable is determined by three energy contributions: (i) the membrane curvature elasticity, controlled by the bending rigidity κ, (ii) the microgel-membrane adhesion energy that arises from the attractive interaction between microgel and membrane, which can be represented as a negative membrane tension $\gamma_{\rm ad}$, and (iii) the microgel compression elasticity determined by the bulk modulus $K_{\rm mg}$.

For a near-spherical shape, the surface deformation can be described by spherical harmonics [17],

$$r(\Omega) = R_{\rm mg}\left[1+\sum_{l,m} u_{lm}Y_{lm}(\Omega)\right] , \qquad \text{(S7)}$$

where Ω is the solid angle. To second order in the deformation amplitudes u_{lm} the bending energy is [17]

$$E_{\rm b} = \frac{\kappa}{2}\sum_{l,m}|u_{lm}|^2\, l(l+1)(l-1)(l+2) \qquad \text{(S8)}$$

The microgel-membrane adhesion energy is

$$\gamma_{\rm ad}A = \gamma_{\rm ad}\Bigg\{4\pi R_{\rm mg}^2(1+u_0)^2 + R_{\rm mg}^2\sum_{l>0}|u_{lm}|^2\left[1+\frac{l(l+1)}{2}\right]\Bigg\} . \qquad \text{(S9)}$$

Finally, for the microgel deformation energy, we consider only the deviations of the actual volume $V = V_{\rm mg}(1+u_0)^3 + R_{\rm mg}^3\sum_{l>0}|u_{lm}|^2$ from the equilibrium volume $V_{\rm mg}$, so that to second order in u_{lm} (for $l>0$)

$$\begin{aligned} E_{\rm mg} &= \frac{K_{\rm mg}}{2}\frac{(V-V_{\rm mg})^2}{V_{\rm mg}} \\ &= K_{\rm mg}\Bigg\{\frac{V_{\rm mg}}{2}\left[(1+u_0)^3-1\right]^2 \\ &\quad - \left[(1+u_0)^3-1\right]R_{\rm mg}^3\sum_{l>0}|u_{lm}|^2\Bigg\} \end{aligned} \qquad \text{(S10)}$$

We determine the amplitude u_0 of the radius variation by minimizing the total energy in the absence of higher-order fluctuation modes, i.e. $u_{lm}=0$ for $l>0$. To obtain analytical expressions for u_0, this minimization is performed by expanding in powers of u_0, so that

$$\gamma_{\rm ad}A^0 = \gamma_{\rm ad}4\pi R_{\rm mg}^2(1+2u_0+u_0^2) \qquad \text{(S11)}$$

and

$$E_{\rm mg}^0 = \frac{K_{\rm mg}}{2}\left(9V_{\rm mg}u_0^2 + 18V_{\rm mg}u_0^3\right) + O[u_0^4] . \qquad \text{(S12)}$$

The energy is then minimal for

$$\begin{aligned} 0 &= \frac{\partial}{\partial u_0}\left[\gamma_{\rm ad}A^0 + E_{\rm mg}^0\right] \\ &= 4\pi R_{\rm mg}^2\left(2\gamma_{\rm ad}\,(1+u_0) + 3K_{\rm mg}R_{\rm mg}u_0\,(1+3u_0)\right) . \end{aligned} \qquad \text{(S13)}$$

To simply the notation, we define

$$|\tilde{\gamma}_{\rm ad}| = -\frac{\gamma_{\rm ad}R_{\rm mg}^2}{8\pi\kappa} \qquad \text{(S14)}$$

$$\tilde{K} = \frac{K_{\rm mg}R_{\rm mg}^3}{8\pi\kappa} . \qquad \text{(S15)}$$

Solving Eq. (S13), we obtain the solution

$$u_0 = \frac{1}{18\tilde{K}}\left[2|\tilde{\gamma}_{\rm ad}| - 3\tilde{K} \pm\sqrt{4|\tilde{\gamma}_{\rm ad}|^2 + 60|\tilde{\gamma}_{\rm ad}|\tilde{K} + 9\tilde{K}^2}\right] \qquad \text{(S16)}$$

for the extrema of the energy. We select the solution corresponding to the energy minimum. To proceed, we consider two limiting cases.

In the limit $|\tilde{\gamma}_{\rm ad}|/\tilde{K} \ll 1$, we obtain from Eq. (S16)

$$u_0 = \frac{2}{3}\frac{|\tilde{\gamma}_{\rm ad}|}{\tilde{K}}\left(1+\frac{4}{3}\frac{|\tilde{\gamma}_{\rm ad}|}{\tilde{K}}\right) \qquad \text{(S17)}$$

In the opposite limit, $\tilde{K}/|\tilde{\gamma}_{\rm ad}| \ll 1$, Eq. (S16) implies

$$u_0 = \frac{2}{3} + \frac{2}{9}\frac{|\tilde{\gamma}_{\rm ad}|}{\tilde{K}} \qquad \text{(S18)}$$

From the total energy

$$E_{\rm total} = E_b + \gamma_{\rm ad}A + E_{\rm mg} \qquad \text{(S19)}$$

with Eqs. (S8), (S9), and

$$E_{\rm mg} = -3u_0K_{\rm mg}R_{\rm mg}^3\sum_{l>0}|u_{lm}|^2 + \mathcal{O}(u_0^2) \qquad \text{(S20)}$$

together with the equipartition theorem, we find the mean-square fluctuation amplitudes

$$\left\langle |u_{lm}|^2\right\rangle = \frac{k_BT}{\kappa}\Big[l(l+1)(l-1)(l+2) - |\tilde{\gamma}_{\rm ad}|\left[l(l+1)+2\right] + 6\tilde{K}u_0\Big]^{-1} . \qquad \text{(S21)}$$

The denominator is positive at large mode numbers l, which implies that the spherical shape is stable with respect to small perturbations for large l. However, the denominator may vanish for small l, which signals a shape instability.

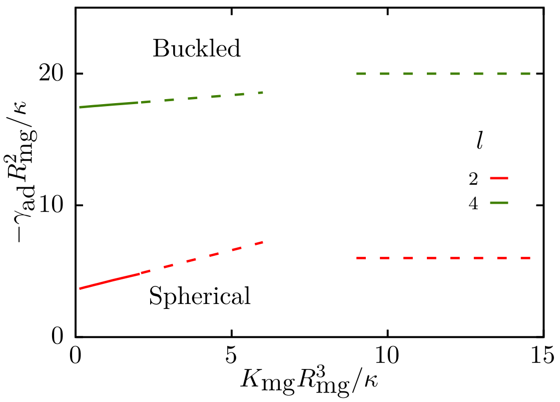

FIG. S19. Buckling condition for $l = 2$ and 4, calculated using Eqs. (S26) and (S31). Buckling occurs at large magnitude of the adhesion-induced membrane tension.

In the case $|\tilde{\gamma}_{ad}|/\tilde{K} \ll 1$, with u_0 from Eq. (S17), we obtain

$$\left\langle |u_{lm}|^2 \right\rangle = \frac{k_B T}{\kappa}\left[A_1 - |\tilde{\gamma}_{ad}|B_1 + C_1\frac{\tilde{\gamma}_{ad}^2}{\tilde{K}}\right]^{-1} \quad \text{(S22)}$$

where

$$A_1 = l(l+1)(l-1)(l+2) \quad \text{(S23)}$$
$$B_1 = l(l+1) - 2 \quad \text{(S24)}$$
$$C_1 = -\frac{16}{3}\,. \quad \text{(S25)}$$

This implies that a low-l modes becomes unstable for physically meaningful solution for the zero of the denominator when

$$|\tilde{\gamma}_{ad}| = \frac{A_1}{B_1} \quad \text{(S26)}$$

In the case $\tilde{K}/|\tilde{\gamma}_{ad}| \ll 1$, with u_0 from Eq. (S18), we find

$$\left\langle |u_{lm}|^2 \right\rangle = \frac{k_B T}{\kappa}\left[A_2 - |\tilde{\gamma}_{ad}|B_2 + C_2\tilde{K}\right]^{-1} \quad \text{(S27)}$$

where

$$A_2 = l(l+1)(l-1)(l+2) \quad \text{(S28)}$$
$$B_2 = l(l+1) + \frac{2}{3} \quad \text{(S29)}$$
$$C_2 = 4\,. \quad \text{(S30)}$$

Thus, the minimal tension for the instability is

$$|\tilde{\gamma}_{ad}| = \frac{A_2}{B_2} + \frac{C_2}{B_2}\tilde{K} \quad \text{(S31)}$$

Figure S19 shows the minimum scaled tension magnitude $|\tilde{\gamma}_{ad}|$ for buckling for the $l = 2$ and 4 modes as a function of the scaled bulk modulus $\tilde{K}$. Our results indicate that a buckled state should occur for a strong adhesion-induced negative tension, a small Young's modulus (low cross-linking) $Y = 3(1-2\nu)K$, and – importantly – a small microgel radius, i.e., for nanogels. The Young's modulus should not be ultralow to prevent the microgel from spreading out on a nearly planar membrane, as the shallow-wrapped state in our phase diagram, see Fig. 3 of the main text.

No simulation values for elastic parameters and tensions are provided in Ref. [16]. Related publications of DPD simulations of membranes and microgels indicate that $K_{mg} \simeq 10\,k_BT/r_c^3$ [18], $\kappa \simeq 100\,k_BT$ [19], and $R_{mg} \simeq 10\,r_c$ [16], where r_c is the interaction range in DPD simulations. This implies that the buckled state observed in Ref. [16], for which we roughly estimate $|\gamma_{ad}| \simeq 10\,k_BT/r_c^2$ [20], i.e., $|\gamma_{ad}R^2_{mg}/\kappa| \simeq 10$, is consistent with our calculation. In contrast, in our simulations $\gamma_{ad} = -N_{lig}U_{eff}/(4\pi R^2_{mg})$ for the transition to the complete-wrapped state, which implies $|\gamma_{ad}R^2_{mg}/\kappa| \simeq 3$, see Fig. 3(a) of the main text, i.e., the spherical shape is stable.

We expect buckling in systems with ligand-receptor mediated adhesion to occur when additional (hidden) ligands in the microgels become available for binding as the microgel is stretched — and the adhesion strength is sufficiently large.

[1] M. Kot, H. Nagahashi, and P. Szymczak, Vis. Comput. **31**, 1339 (2015).
[2] G. Gompper and D. M. Kroll, in *Statistical Mechanics of Membranes and Surfaces* (WORLD SCIENTIFIC, 2004) pp. 359–426.
[3] D. M. Kroll and G. Gompper, Science **255**, 968 (1992).
[4] K. A. Brakke, Exp. Math. **1**, 141 (1992).
[5] S. Dasgupta, T. Auth, and G. Gompper, Soft Matter **9**, 5473 (2013).
[6] S. Dasgupta, T. Auth, and G. Gompper, Nano Lett. **14**, 687 (2014).
[7] S. Dasgupta, T. Auth, N. S. Gov, T. J. Satchwell, E. Hanssen, E. S. Zuccala, D. T. Riglar, A. M. Toye, T. Betz, J. Baum, and G. Gompper, Biophys. J. **107**, 43 (2014).
[8] G. A. Vliegenthart and G. Gompper, New J. Phys. **13**, 045020 (2011).
[9] K. L. Johnson, *Contact mechanics*, 9th ed. (Cambridge University Press, Cambridge, 2003).
[10] G. San-Vicente, I. Aguinaga, and J. T. Celigueta, IEEE Trans. Visual. Comput. Graphics **18**, 228 (2012).
[11] A. J. Ladd and J. H. Kinney, Physica A **240**, 349 (1997).

[12] M. Deserno, Phys. Rev. E **69**, 031903 (2004).
[13] J. Liam McWhirter, H. Noguchi, and G. Gompper, New J. Phys. **14**, 085026 (2012).
[14] J. Rudnick and G. Gaspari, J. Phys. A: Math. Gen. **19**, L191 (1986).
[15] J. Aronovitz and D. Nelson, J. Phys. France **47**, 1445 (1986).
[16] P. Desai, R. Rimal, A. Florea, R. A. Gumerov, M. Santi, A. S. Sorokina, S. E. M. Sahnoun, T. Fischer, F. M. Mottaghy, A. Morgenroth, A. Mourran, I. I. Potemkin, M. Möller, and S. Singh, Angew. Chem. Int. Ed. **61**, e202116653 (2022).
[17] S. T. Milner and S. A. Safran, Phys. Rev. A **36**, 4371 (1987).
[18] S. V. Nikolov, A. Fernandez-Nieves, and A. Alexeev, Proc. Natl. Acad. Sci. U.S.A. **117**, 27096 (2020).
[19] J. C. Shillcock and R. Lipowsky, J. Chem. Phys. **117**, 5048 (2002).
[20] N. V. Bushuev, R. A. Gumerov, S. Bochenek, A. Pich, W. Richtering, and I. I. Potemkin, ACS Appl. Mater. Interfaces **12**, 19903 (2020).
